# CARDIAC MYBP-C IN C57BL/6 MICE: THE EFFECTS OF AGE

**DOI:** 10.1093/geroni/igz038.3301

**Published:** 2019-11-08

**Authors:** Amanda Pinheiro, Ted G Graber, LaDora V Thompson

**Affiliations:** 1 Boston University, Boston, Massachusetts, United States; 2 East Carolina University, Department of Physical Therapy, Greenville, North Carolina, United States

## Abstract

Aging is a known contributor to cardiovascular dysfunction. It is well-established that with age there are functional changes in the heart; yet, the proteins responsible for maintaining sarcomere integrity are not well understood during the aging process. A key protein, cardiac myosin binding protein C (cMyBP-C), contributes to the structural integrity and the regulation of actomyosin interactions. To date, little is known about the effects of aging on cMyBP-C. Therefore, the first step in evaluating this sarcomere protein was to determine the expression of cMyBP-C in cardiac tissue across the lifespan. Using ten C57BL/6 male mice per age group (adult (6-7 months), old (22-25 months), and very old (≥29 months)), body and heart mass were determined. Next a portion of the cardiac tissue was homogenized, and protein concentration was determined (BCA assay). The protein samples were probed for cMyBP-C with MYBPC3 (Abcam, #ab133499) by Western Blot. One-way ANOVA was performed to evaluate differences between groups. Results indicated there was an increase in heart mass with age, but relative to body weight there was no significant difference between the three age groups. Western blot analysis revealed no significant age-related difference in the expression of cMyBP-C. Although there was no change in expression levels, it is not possible to rule out cMyBP-C as a contributor to age-related cardiac dysfunction because phosphorylation is known to play a critical role in the function of cMyBP-C. Thus, further investigation of the phosphorylation status of cMyBP-C is needed and is ongoing.

